# Comparison of laparoscopic and open pancreaticoduodenectomy for the treatment of distal cholangiocarcinoma: A propensity score matching analysis

**DOI:** 10.3389/fonc.2022.1057337

**Published:** 2022-11-18

**Authors:** Yuwen Zhu, Guangchen Zu, Di Wu, Yue Zhang, Yang Yang, Han Wu, Xuemin Chen, Weibo Chen

**Affiliations:** ^1^ Department of Hepatopancreatobiliary Surgery, The Third Affiliated Hospital of Soochow University, Changzhou, China; ^2^ Department of Hepatobiliary Surgery, Eastern Hepatobiliary Surgery Hospital, Second Military Medical University (Naval Medical University), Shanghai, China

**Keywords:** distal cholangiocarcinoma, laparoscopic pancreaticoduodenectomy, survival, propensity score matching, prognostic factors

## Abstract

**Background:**

There are few studies comparing the oncological outcomes of laparoscopic pancreaticoduodenectomy (LPD) and open pancreaticoduodenectomy (OPD) for distal cholangiocarcinoma (DCC). Our objective was to assess the short-term efficacy and long-term survival of LPD and OPD in patients with DCC.

**Methods:**

The data of 124 DCC patients who underwent LPD or OPD at the Third Affiliated Hospital of Soochow University from May 2010 to May 2021 were retrospectively analyzed. Propensity score matching was performed to balance the two groups of baseline characteristics. After 1:1 matching, the overall survival (OS) of the two groups was compared by the Kaplan−Meier method. Univariate and multivariate Cox regression analyses were used to identify independent predictors of OS.

**Results:**

The original cohort consisted of 124 patients. Nineteen patients were excluded because of incomplete baseline or follow-up data, and the remaining 105 patients were divided into two cohorts (45 in the LPD group and 60 in the OPD group). The LPD group showed more favorable results in OS analysis (LPD vs. OPD, 56.4 [46.2-66.5] vs. 48.9 [36.4-61.4], months, P=0. 01). PSM analysis identified 30 pairs of patients, and differences between matching groups were still significant (LPD vs. OPD, 67.9[58.2-77.6] vs. 47.4[31.4-67.5], months, P=0.002). Moreover, the LPD group experienced less intraoperative bleeding (LPD vs. OPD, 292.67 vs. 519.17 mL, P=0.002). Univariate analysis showed that surgical modality (P=0.012), carbohydrate antigen 19-9 (P=0.043), carcinoembryonic antigen (P=0.003), neutrophil-to-lymphocyte ratio (P=0.012), blood transfusion (P=0.031), clinically relevant postoperative pancreatic fistula (P<0.001) and lymphatic metastasis (P=0.004) were predictors of OS. Multivariate Cox analysis demonstrated that carbohydrate antigen 19-9 (P=0.048), carcinoembryonic antigen (P=0.031) and lymphatic metastasis (P=0.023) were independent predictive factors of OS. However, adjuvant therapy had no significant effect on the OS of DCC patients after radical pancreaticoduodenectomy (P>0.05).

**Conclusions:**

For DCC patients, LPD may be a more recommended procedure because of its advantages over OPD in terms of intraoperative bleeding and long-term survival.

## Introduction

Distal cholangiocarcinoma is an epithelial malignancy originating in the middle and lower segments of the common bile duct and the ampulla of Vater (1), which accounts for approximately 20%-40% of cholangiocarcinoma (2). DCC is highly malignant and has a poor prognosis. The 1-year, 3-year and 5-year OS rates are 46%, 18% and 11%, respectively (3). Old age among males and chronic biliary tract disease are potential risk factors for its occurrence (4). Lymphatic metastasis and nerve infiltration are the main modes of invasion. Pancreaticoduodenectomy is the normative and sole therapeutic method for DCC patients (5, 6). The first case of laparoscopic pancreaticoduodenectomy (LPD) was reported by Gagner et al. in 1994 (7, 8) and has been widely carried out worldwide since then. In the past two decades, LPD has been widely used to treat DCC (3). With the advancement of operative techniques and perioperative care, the postoperative survival rate of DCC patients has been significantly improved (9). However, it is still unclear whether LPD is superior to OPD in terms of short-term outcomes and long-term survival (10). Some studies suggest that LPD takes a long time, has a complicated operation and is high risk, which has high requirements for the surgical team and a high incidence of postoperative complications (11, 12). Several recent multicenter studies have shown that LPD is a secure and practical approach. In high-volume centers with sufficient surgical experience, LPD appears to be an effective alternative, which is associated with a shorter hospital stay and similar short-term morbidity and mortality to OPD. Nevertheless, despite extensive procedural expertise, the clinical benefits of LPD compared to OPD are still insignificant (13–16). However, few studies have focused on LPD and OPD in DCC, so we conducted this study to focus on the differences between LPD and OPD in DCC to help guide the surgical treatment of DCC.

## Methods

### Study design and patient selection

The data of DCC patients who underwent pancreatoduodenectomy at the Third Affiliated Hospital of Soochow University from May 2010 to May 2021 were retrospectively analyzed. The inclusion criteria were as follows: 1. all patients underwent PD radical surgery; 2. postoperative pathological examination confirmed distal cholangiocarcinoma; 3. preoperative imaging examination showed that the tumor had no distant metastasis; and 4. there was no other malignant tumor resection history.

The exclusion criteria were as follows: 1. incomplete clinical records or loss during follow-up; 2. patients who received neoadjuvant therapy before surgery; 3. patients with severe underlying disease who could not tolerate surgery; and 4. patients only received palliative treatment. The surgeon explained the procedure of pancreaticoduodenectomy clearly.

### Data collection

We obtained patient demographics, laboratory data, postoperative pathological results and follow-up data from the medical records database. Preoperative data consisted of age, body mass index (BMI), smoking history, sex, height, history of diabetes, history of hypertension, American Society of Anesthesiologists (ASA) score (17) and tumor markers, including carbohydrate antigen 19-9 (CA19-9), carbohydrate antigen 125 (CA125) and carcinoembryonic antigen (CEA). Liver function biochemical data and routine blood indicators, such as platelets, total bilirubin, neutrophil (N) count, lymphocyte (L) count, neutrophil-to-lymphocyte ratio (NLR) and systemic immune inflammation index (SIII=P*N/L), were also calculated (17).

Intraoperative observation indicators included the harvested lymphatic nodes, intraoperative bleeding volume and blood transfusion (obtained through surgical records and anesthesia records). Pathological results included differentiation degree, R0 resection, lymphatic metastasis and tumor stage. The tumor stages were determined according to the 8th edition of the DCC TNM staging definition proposed by the American Joint Committee on Cancer (AJCC) and the Union for International Cancer Control (UICC) in 2018 (18). The depth of tumor invasion and lymphatic metastasis were recorded in every patient.

Postoperative observation data included hospitalization time after operation, postoperative complications, postoperative adjuvant therapy and overall survival. Postoperative hospital stay was defined as the number of days from surgery to discharge. Postoperative complications were classified according to the Clavien−Dindo (CD) classification (19), including abdominal infection, clinically relevant postoperative pancreatic fistula (CR-POPF), and delayed gastric emptying (DGE) (17). According to the International Research Group on Pancreatic Surgery (ISGPS) definition, in the current study, only grade B and C POPF were thought to be complications, and the previously defined grade A biochemical pancreatic fistula report was no longer considered a clinical complication (20, 21). OS was defined as the duration from surgical resection to clinical death or last follow-up. To ensure adequate follow-up time for survival analysis, patients who received PD after May 2021 were excluded. Postoperative follow-up was conducted by outpatient visits, inpatient medical record systems and telephone calls. The follow-up period lasted until the end of May 2022 or the patient’s death.

### Propensity score matching

In recent years, an increasing number of surgical studies have begun to apply PSM analysis to effectively reduce the influence of confounding factors in the study (22). In this study, two groups were matched by propensity score matching. Age, sex, BMI, diabetes, smoking history, ASA score and clinical laboratory test data, including CA125, CEA, platelets, total bilirubin, neutrophil count, lymphocyte count, SIII and other factors related to surgical or postoperative management, were selected as matching factors. Next, PSM was performed at 1:1. After matching, 45 patients were excluded. The LPD group included 30 patients, and the OPD group included 30 patients, for a total of 60 patients. To prevent prognostic factors such as adjuvant therapy and R0 resection from affecting the construction of the propensity score model, only baseline variables were included.

### Statistical analyses

For the entire cohort, categorical variables were expressed as frequencies and percentages (%) and compared using the chi-square test (X^2^) or Fisher’s exact test. Continuous variables conforming to a normal distribution were calculated as the mean ± standard deviation (SD) and compared by Student’s t test. Continuous variables with a nonnormal distribution are represented by the median and interquartile range (IQR), and the differences were compared by the Mann−Whitney U test. The Kaplan−Meier method was adopted to draw survival curves, and the log-rank test was used for univariate analysis of the clinicopathological factors associated with OS. Factors showing statistically significant differences in univariate analysis were included in multivariate Cox regression analysis, to determine the independent risk factors affecting the prognosis of patients. P<0.05 was considered to indicate statistical significance. All statistical analyses were performed by using IBM SPSS Statistics (version 26.0, IBM Corp).

## Results

### The baseline data

**Figure 1:**
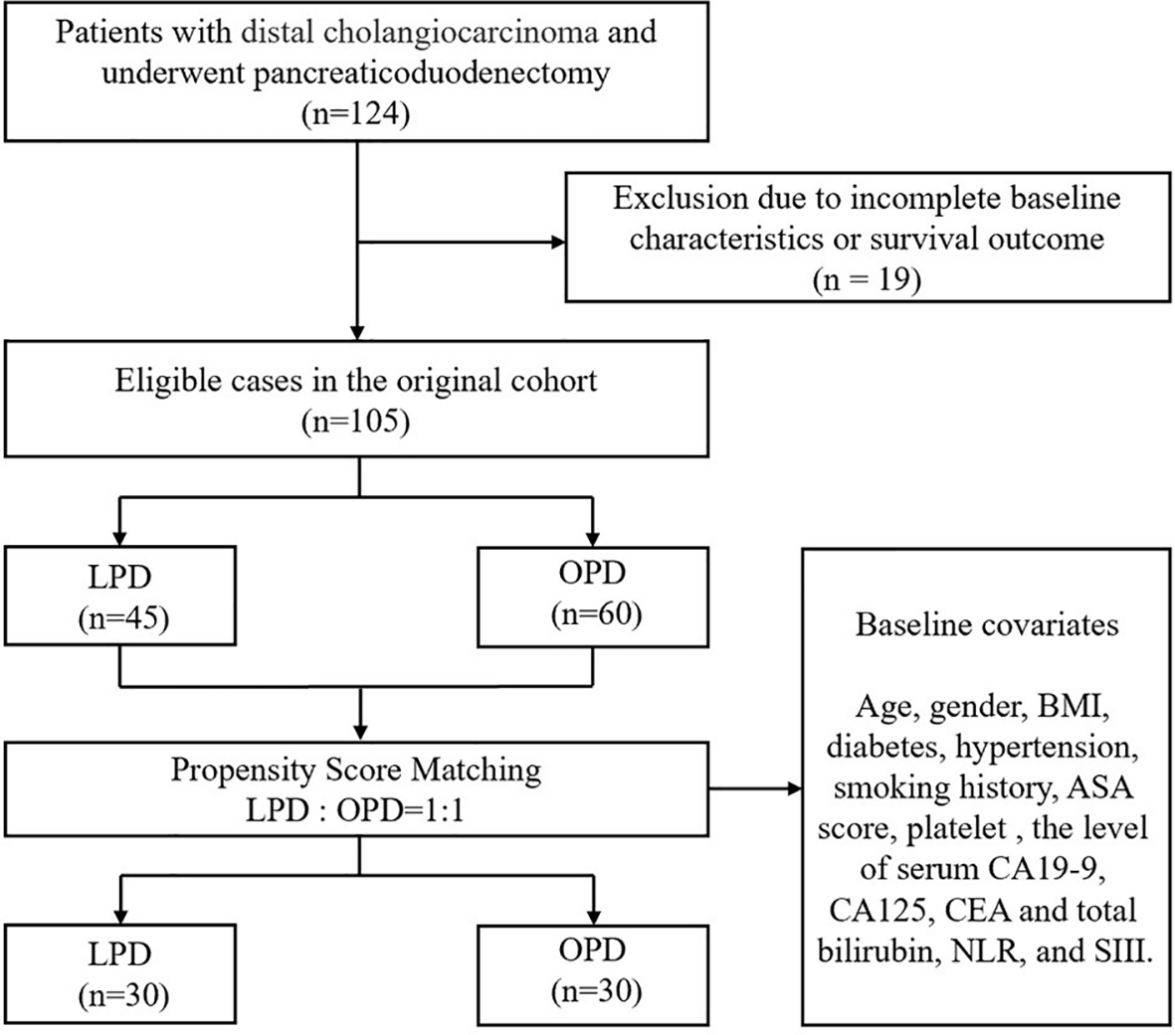
Flow chart of patient selection.

As shown in **Figure 1**, 124 people with DCC who received PD were enrolled in the study. Nineteen patients were excluded because of incomplete baseline or follow-up data, and the remaining 105 patients were divided into two cohorts (45 in LPD and 60 in OPD). Covariates such as sex, age, BMI, comorbidities, ASA, TNM stage, and preoperative tumor markers were included in the 1:1 PSM analysis. After PSM analysis, 30 LPD patients (66.7%) and 30 OPD patients (50.0%) were matched. Baseline characteristics were equilibrated to reduce the impact of confounding factors between the two groups of patients on the study.

The mean age was 65.3 ± 7.7 years in the LPD group and 63.1 ± 9.9 years in the OPD group (P=0.232). The proportion of male patients was 82.2% in the LPD group and 48.3% in the OPD group (P=0.804). There was no statistical significance before and after PSM (P>0.05). The two teams had similarities in BMI and comorbidities. Furthermore, there was no statistical significance in total bilirubin, platelet, NLR, SIII or preoperative cancer biomarkers including CA19-9, CA125 and CEA, between the two groups, as detailed in **Table 1**.

**Table 1:** Comparisons of patients’ clinicopathologic characteristics between patients with DCC underwent LPD and OPD before and after propensity score matching.

Variables	Before PSM			After PSM (1:1)		
	LPD (n=45)	OPD (n=60)	P value	LPD (n=30)	OPD (n=30)	P value
Age	65.3	63.1	0.232	64.1	64.3	0.936
Male	29 (64.4)	37 (61.7)	0.840	17 (56.7)	19 (63.3)	0.792
BMI	22.9	22.1	0.151	23.4	22.2	0.088
Diabetes mellitus	7 (15.6)	8 (13.3)	0.784	5 (16.7)	5 (16.7)	0.635
Smoke	6 (13.3)	8 (13.3)	0.617	3 (10.0)	4 (13.3)	0.500
Hypertension	19 (42.2)	20 (33.3)	0.416	13 (43.3)	10 (33.3)	0.596
CA19-9 (U/mL)	188.5	149.6	0.430	192.1	162.9	0.677
CA125 (U/mL)	21.1	17.8	0.355	22.7	17.3	0.310
CEA (ng/mL)	4.2	3.9	0.789	4.0	3.7	0.798
Platelet (10^9^/L)	236.2	224.5	0.557	223.0	211.0	0.480
TBil (μmol/L)	106.3	141.6	0.107	112.0	131.6	0.512
Neutrophil (10^9^/L)	4.1	4.6	0.325	3.9	4.8	0.111
NLR	3.8	4.7	0.219	3.8	5.1	0.205
SIII	904.8	1053.2	0.378	844.7	1079.1	0.268
TNM stage (IIB)	45	36	0.250	24	27	0.472
Blood transfusion	12 (26.7)	24 (40.0)	0.213	9 (30.0)	10 (33.3)	0.500
ASA score	2.2	2.2	0.813	2.23	2.27	0.802

BMI, body mass index; CA19-9, carcinoembryonic antigen 19-9; CA125, carcinoembryonic antigen 125; CEA, carcinoembryonic antigen; TBil, total bilirubin; NLR, neutrophil to lymphocyte ratio; SIII, systemic-immune-inflammation Index; ASA, American Society of Anesthesiologists score.

### Clinicopathological and postoperative characteristics

As demonstrated in **Table 2**, the intraoperative bleeding in the LPD group was less than that in the OPD group (mean, 292.67 vs. 519.17 mL, P=0.002), and less intraoperative blood transfusion was required (mean, 591.67 vs. 880.00 mL, P=0.033). Although the LPD group had more harvested lymph nodes in the original cohort (mean, 14.58 vs. 10.20, P=0.01), there was no statistical significance after PSM analysis (mean, 14.3 vs. 9.93, P=0.076). Other surgical results and pathological features, such as lymphatic metastasis, R0 resection and AJCC TNM stage, were not significantly different between the two matched groups.

**Table 2:** Comparisons of clinical outcomes between patients with LPD and OPD before and after propensity score matching.

Variables	Before PSM			After PSM		
	LPD	OPD	P value	LPD	OPD	P value
**Perioperative surgeical results**						
lymphatic metastasis	0.51	1.20	0.055	0.07	0.33	0.073
Harvested lymph nodes	14.58	10.20	**0.010**	14.30	9.93	0.076
Intraoperative bleeding	292.67	519.17	**0.002**	302.33	505.00	**0.030**
blood transfusion volume	591.67	880.00	**0.033**	570.00	967.50	**0.042**
R0 Resection	41 (91.1)	56 (93.3)	0.675	28 (93.3)	28 (93.3)	1.000
abdominal infection	7 (15.6)	6 (10.0)	0.551	5 (16.7)	4 (13.3)	0.718
CR-POPF	10 (22.2)	14 (23.3)	0.895	8 (26.7)	8 (26.7)	1.000
DGE	3 (6.7)	7 (11.7)	0.393	2 (6.7)	4 (13.3)	0.398
Postoperative stay	23.89	23.20	0.828	25.10	24.03	0.817
Adjuvant treatment	16 (35.6)	20 (33.3)	0.815	8 (26.7)	9 (30.0)	0.779
Mortality during the follow-up	14 (31.1)	45 (75.0)	**<0.001**	5 (8.3)	22 (73.3)	**<0.001**
**OS**	56.4	48.9	**0.010**	67.9	47.4	**0.002**
1 year OS rate (%)	84.4	78.3		83.2	76.7	
2 year OS rate (%)	74.9	55.0		83.2	53.3	
3 year OS rate (%)	62.4	41.3		–	–	

CR-POPF, clinically relevant postoperative pancreatic fistula; DGE, clinically relevant delayed gastric emptying; OS, overall survival. bold value means P<0.05.

As two sets of postoperative data showed, there was no statistical significance in hospitalization time after operation, DGE, CR-POPF or intra-abdominal infection between the two teams (P>0.05).

### Survival analysis

**Figure 2:**
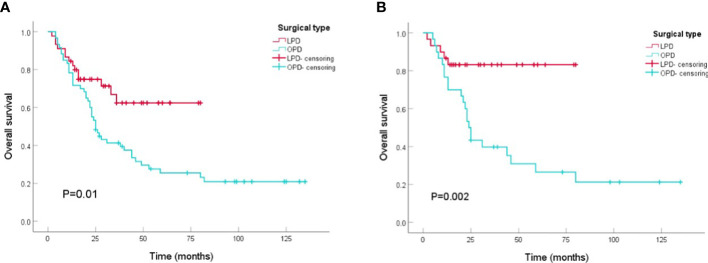
Kaplan-Meier curves of overall survival between patients with OPD and LPD. **(A)** Kaplan-Meier curves of overall survival between patients with OPD and LPD in the original cohort. **(B)** Kaplan-Meier curves of overall survival between patients with OPD and LPD in the PSM cohort.

Patients who underwent PD from 2010 to 2021 (n=105) were included in the survival analysis. Comparing the tumor prognosis of the two groups, the LPD group had a lower mortality during the follow-up (31.1 vs. 75.0%, P<0.001) and showed preferable results in OS analysis (LPD vs. OPD, 56.4[46.2-66.5] vs. 48.9[36.4-61.4], months, P=0. 01) (**Figure 2A**). After PSM analysis, the difference in mortality during the follow-up period was still marked (8.3 vs. 73.3%, P<0.001), and the OS benefit of LPD was still superior to that of OPD (LPD vs. OPD, 67.9[58.2-77.6] vs. 47.4[31.4-67.5], months, P=0. 002) (**Figure 2B**).

### Univariate analysis of prognosis

Univariate Cox regression analysis indicated that the surgical approach was a predictor of overall survival (HR=0.463[0.253-0.847], P=0.012). Other OS-related factors included CA19-9 (HR=2.561[1.030-6.369], P=0.043), CEA (HR=4.095[1.639-10.232], P=0.003), NLR (HR=2.885[1.258-6.620], P=0.012), blood transfusion (HR=1.768[1.053-2.967], P=0.031), CR-POPF (HR=0.249[0.124-0.500], P<0.001), and lymphatic metastasis (HR=1.170[1.052-1.302], P=0.004). Sex, BMI, complications and abdominal infection were not important prognostic factors (**Table 3**).

**Table 3:** Independent prognostic factors of OS by Cox-regression analysis of the whole cohort before and after the PSM.

Variables	Univariate Analysis		Multivariate Analysis	
	UV HR (95%CI)	P	MV HR (95%CI)	P
Surgical Modality (LPD vs OPD)	0.463 (0.253-0.847)	**0.012**	0.259 (0.036-1.874)	0.181
Age	1.029 (0.999-1.060)	0.059	0.996 (0.882-1.125)	0.950
Male	1.432 (0.829-2.474)	0.198		
BMI	0.936 (0.854-1.027)	0.163		
Diabetes mellitus	1.128 (0.535-2.379)	0.752		
Smoke	0.738 (0.335-1.626)	0.451		
Hypertension	1.135 (0.669-1.926)	0.639		
CA19-9 (U/mL) (>37)	2.561 (1.030-6.369)	**0.043**	2.928 (1.401-21.354)	**0.048**
CA125 (U/mL) (>35)	2.250 (0.837-6.043)	0.108		
CEA (ng/mL) (>5)	4.095 (1.639-10.232)	**0.003**	9.404 (2.240-39.196)	**0.031**
TBil (μmol/L) (>100)	1.568 (0.735-3.346)	0.245		
Neu/lymph ratio (>3)	2.885 (1.258-6.620)	**0.012**	1.095 (0.052-23.130)	0.953
SIII (>900)	1.548 (0.705-3.396)	0.276		
TNM Stage IIA	0.220 (0.030-1.625)	0.138		
Blood transfusion	1.768 (1.053-2.967)	**0.031**	1.431 (1.100-20.498)	0.792
Intraoperative bleeding (>500)	0.484 (0.137-1.708)	0.259		
CR-POPF	0.249 (0.124-0.500)	**<0.001**	1.795 (0.536-6.008)	0.625
ASA score	1.538 (0.895-2.642)	0.119		
Lymphatic metastasis	1.170 (1.052-1.302)	**0.004**	1.822 (1.203-3.321)	**0.023**
abdominal infection	1.376 (0.675-2.807)	0.380		
DGE	1.688 (1.777-3.688)	0.186		
Adjuvant treatment	1.263(0.737-2.166)	0.343		

BMI, body mass index; CA19-9, carcinoembryonic antigen 19-9; CA125, carcinoembryonic antigen 125; CEA, carcinoembryonic antigen; TBil, total bilirubin; NLR, neutrophil to lymphocyte ratio; SIII, systemic-immune-inflammation Index; CR-POPF, clinically relevant postoperative pancreatic fistula; ASA, American Society of Anesthesiologists score; DGE, clinically relevant delayed gastric emptying; bold value means P<0.05.

### Multivariate analysis of predictors

The OS-related factors with a P value less than 0.1 in the univariate analysis were included in the multivariate analysis, which indicated that age, surgical method, blood transfusion, CR-POPF and NLR were no longer prognostic factors. CA19-9 (HR=2.928[1.401-21.354], P=0.048), CEA (HR=9.404[2.240-39.196], P=0.031) and lymphatic metastasis (HR=1.822[1.203-3.321], P=0.023) were still markedly related to OS, while postoperative adjuvant chemotherapy had no significant influence on OS (P=0.343) (**Table 3**).

## Discussion

DCC is highly malignant and prone to lymphatic metastasis or nerve infiltration, and the prognosis is poor (4). At present, PD is the sole feasible and effective method for the treatment of resectable DCC. To reach the purpose of radical resection, lymph nodes need to be carefully cleaned to ensure R0 resection in PD (23). With the continuous progress of surgical technology, LPD has become a routine procedure in some pancreatic centers in recent years (24). Some studies have reported that LPD has a shorter hospital stay and rapid postoperative recovery and is not inferior to OPD in short-term oncology results (25, 26). However, there are still few studies on the treatment of DCC with LPD and OPD. This study found that the LPD group achieved similar clinical outcomes in terms of surgical safety and radical effects, and the LPD group had better long-term survival than OPD.

The incidence of total complications following LPD and OPD was reported to be comparable. In the LPD group and the OPD group, the incidence of CR-POPF was 18.02% and 18.73%, respectively, while the incidence of intra-abdominal infection was 10% and 11% (15, 27). Our study indicated that the average intraoperative bleeding volume in the LPD group was less than that in the OPD group (mean, 292.67 vs. 519. 17 mL, P=0.002), and fewer intraoperative blood transfusions were required (mean, 591.67 vs. 880. 00 mL, P=0.033). This may be due to laparoscopic amplification and clearer vision; thus, the intraoperative vascular exposure could be conducted, and intraoperative bleeding could be managed more clearly and precisely. The data in this study showed that the rate of CR-POPF in the LPD group and the OPD group was 22.2% and 23.3%, respectively, and the incidence of intra-abdominal infection was 15.6% and 10.0%, respectively. There was no statistical significance (P>0.05) and similar to the results reported in the literature, indicating that LPD can achieve short-term efficacy similar to OPD for DCC.

In addition, to achieve the consequence of radical resection, R0 resection is required (23). There was no statistical significance in R0 resection between LPD and OPD (91.1% vs. 93.3%, P=0.675). It is shown that LPD and OPD can achieve similar results in R0 resection, and it is consistent with Boggi et al. ‘‘s report that the R0 resection rate of LPD for tumors can reach 73%~100%. As distal cholangiocarcinoma is prone to lymphatic metastasis, it is widely accepted that lymphatic metastasis is an independent risk factor affecting the prognosis of DCC patients (28). In this research, we also found that lymphatic metastasis was a prognostic factor for the OS of DCC patients (P<0.05). Although research has pointed out that lymphatic metastasis and harvested lymph nodes are prognostic factors for DCC (29), there are still few studies on the relationship between harvested lymph node number and the long-term survival of DCC. In our study, although the LPD group had more harvested lymph nodes in the original cohort (mean, 14.58 vs. 10.20, P=0.01), there was no statistical significance after PSM analysis (mean, 14.3 vs. 9.93, P=0.076). This reveals that the two teams are similar in technical feasibility.

Several studies have shown that LPD and OPD have similar long-term survival rates in the treatment of pancreatic and periampullary cancers (15, 27). A recently published paper showed that there was no significant difference in long-term survival between LPD and OPD in DCC (30). In contrast, our research revealed that the prognosis for LPD was improved. Comparing the potential causes of the different outcomes, although there was no statistically significant difference after PSM, LPD harvested more lymph nodes in the initial cohort, which may be worthy of further study. Second, research has demonstrated that blood transfusions have an impact on PD patients’ long-term survival after surgery (31). Our results supported this finding and showed that LPD patients receive fewer blood transfusions, which may help improve the prognosis for LPD patients.

Many studies have focused upon the adjuvant treatment of DCC (32, 33) because the tumor heterogeneity of DCC leads to poor targeted therapy for this disease. At present, the use of gemcitabine combined with platinum drugs is generally accepted internationally, and the existence of positive lymph is an indication of adjuvant therapy (34, 35). We found that adjuvant therapy was not a predictor of OS, and the proportion of postoperative adjuvant therapy in DCC patients was 34.3%, which was lower than the international average (36). The negative results might be due to the limited numbers of samples who received postoperative adjuvant therapy in the research, and multicenter studies on DCC adjuvant therapy after PD are expected.

The study still has some limitations. First, this is a single-center, small-sample retrospective study and may inevitably involve residual confounding factors. Second, because patients do not receive unified treatment, there may be disunity factors that affect the survival of patients. To provide a clearer conclusion on the LPD of DCC, a massive prospective randomized controlled trial is needed. Hopefully soon, we can conduct a multicenter randomized trial.

In summary, compared with OPD, LPD significantly reduced intraoperative bleeding volume and blood transfusion in DCC treatment and showed a similar postoperative complication rate. With better long-term survival outcomes than OPD, LPD can be a preferred surgical option for DCC patients.

## Data availability statement

The original contributions presented in the study are included in the article/supplementary material. Further inquiries can be directed to the corresponding authors.

## Ethics statement

The studies involving human participants were reviewed and approved by Third Affiliated Hospital of Soochow University. The patients provided their written informed consent to participate in this study.

## Author contributions

WC and XC designed the research and revised the manuscript. XC, WC, YueZ performed the surgery with the help of their colleagues. YuwZ, GZ, DW gathered results of each patient. YueZ and HW analyzed the data. YuwZ wrote the manuscript. All authors contributed to the article and approved the submitted version.

## Funding

This study was supported by Jiangsu “333” high-level talent Project (2022, 3-4-086), Changzhou Health High-level Top-notch Talent Project (2022), Changzhou Science and technology support plan for social development (No. CE20225043). Young Talent Development Plan of Changzhou Health Commission (No. CZQM2020005, No. CZQM2021002), Major Science and Technology Project of Changzhou Health Commission (ZD201906), Applied Basic Research of Changzhou Technology Bureau (CJ20190093), and Young Talent Science and Technology Project of Changzhou Health Commission (No. QN202101).

## Conflict of interest

The authors declare that the research was conducted in the absence of any commercial or financial relationships that could be construed as a potential conflict of interest.

## Publisher’s note

All claims expressed in this article are solely those of the authors and do not necessarily represent those of their affiliated organizations, or those of the publisher, the editors and the reviewers. Any product that may be evaluated in this article, or claim that may be made by its manufacturer, is not guaranteed or endorsed by the publisher.
